# Supplementary material for: MetaRibo-Seq measures translation in microbiomes
Source: Nat Commun. 2020 Jun 29;11:3268. doi: 10.1038/s41467-020-17081-z (PMC7324362; doi:10.1038/s41467-020-17081-z)
Supplement: Supplementary file 10 — Supplementary Data 7 [file 41467_2020_17081_MOESM10_ESM.zip › File2/Confidence_High_Taxonomy/40371_out.krona.html]

Javascript must be enabled to view this page.

members
magnitude
magnitudeUnassigned
count
unassigned
taxon
rank

40371\_out

3

superkingdom
3
2

phylum
3
976

200643
3
class

order
171549
3

3
171552
family

genus
3
838

165179
3

SRS023715\_contig\_number\_9740SRS049896\_contig\_number\_910SRS1041116\_contig\_number\_1841
species
